# Supplementary material for: Team pro-social rule breaking and team innovation performance: An information processing theory perspective
Source: PLoS One. 2024 Jul 11;19(7):e0303800. doi: 10.1371/journal.pone.0303800 (PMC11239028; doi:10.1371/journal.pone.0303800)
Supplement: Attachment — Submitted filename: Response to Reviewers.docx [file pone.0303800.s001.docx]

Please use the space provided to explain your answers to the questions above. You may also include additional comments for the author, including concerns about dual publication, research ethics, or publication ethics. (Please upload your review as an attachment if it exceeds 20,000 characters)

Reviewer #1: The objective of the paper " Team pro-social rule breaking and team innovation performance: An information processing theory perspective" is interesting but the current version of manuscript needs a radical new structure and development.
First, the aim should be evidenced in the abstract and introduction sections.

Our response: Thanks very much for your helpful suggestions. We have optimized the abstract and introduction sections to highlight the main purpose of this paper. Please refer to the respective sections in the revised manuscript for details (Line 30-31, 43-45, 59-61, 65-67).

Second, the methodological approach should be explained in a clearer way.

Our response: Thanks very much for your helpful suggestions. We have added a description of the data aggregation process for TPSRB in the methods section, along with explanations of variable reliability and validity testing. To ensure clarity in describing the methodology, we have included an overview of the method's implementation process in the methods and data analysis sections. Please refer to the specific sections for detailed information (Line 361-370, 382-392,).

Third, the contribution can be made evident only putting the accent on the gap in the literature.

Our response: Thanks very much for your helpful suggestions. We acknowledge that the limitations in the paper's contributions stemmed from not adequately emphasizing the gaps in the literature. To address this issue, members of the research team reevaluated relevant literature and engaged in focused discussions on PSRB, team reflexivity and team learning oriented papers. Based on a review of past literature, we identified the main gaps in the following areas: 1. Research on PSRB primarily focuses on the individual level, with results mostly revealing the negative effects of PSRB on individual performance evaluations. There is a lack of research on PSRB at the team level and exploration of its positive effects. 2. Studies on factors influencing team reflexivity have concentrated more on organizational contextual factors and team member traits, with a shortage of investigations into how team behavioral tendencies impact the team reflexivity mechanism. 3. Research on team learning orientation has predominantly focused on its direct effects on organizational outcomes, with limited studies on the interaction effects of team learning orientation with other organizational characteristics in complex organizational contexts. This paper reexamines these identified gaps and builds upon them to formulate the paper's contributions. For specific details, please refer to the Introduction section (line 79 to 97).

Finally, the interpretation of results are not consistent with the objectives and need further improvement also in terms of policy implications.

Our response: Thanks very much for your helpful suggestions. We conducted a reanalysis of the data analysis results and summarized the support for our hypotheses. According to the results of hierarchical regression analysis, the interaction between team learning orientation and TPSRB has a positive direct effect on team reflexivity, consistent with Hypothesis 1. Additionally, the results support the positive role of team reflexivity in team innovation performance, aligning with Hypothesis 2. Finally, we examined the moderated mediation effect, and the results indicated that only under high levels of team learning orientation, TPSRB's mediated effect on team innovation performance through team reflexivity is established, in line with Hypothesis 3. Furthermore, based on the findings of this study, we have updated the managerial implications, emphasizing how managers in complex organizational contexts can enhance employee innovation performance by comprehensively understanding and promoting TPSRB. For specific details, please refer to the specific sections (Line 522-524).

Reviewer #2: Authors have done wonderful work appreciation to the authors. There few concerns which authors need to incorporate
1. Add Factor loadings table

Our response: Thanks very much for your helpful suggestions. Your suggestions have helped us improve the rigor of our data analysis. We have added a factor loading table, CR and AVE in the data analysis chapter. The specific content is as follows, and you can also review the details in the data analysis chapter (line 361 to 371).

| Variable | items | loadings | CR | AVE |
| --- | --- | --- | --- | --- |
| team pro-social rule breaking | item1 | 0.587 | 0.927 | 0.496 |
|  | item2 | 0.662 |  |  |
|  | item3 | 0.737 |  |  |
|  | item4 | 0.652 |  |  |
|  | item5 | 0.598 |  |  |
|  | item6 | 0.645 |  |  |
|  | item7 | 0.713 |  |  |
|  | item8 | 0.784 |  |  |
|  | item9 | 0.761 |  |  |
|  | item10 | 0.787 |  |  |
|  | item11 | 0.78 |  |  |
|  | item12 | 0.704 |  |  |
|  | item13 | 0.702 |  |  |
| team reflexivity | item1 | 0.81 | 0.942 | 0.598 |
|  | item2 | 0.852 |  |  |
|  | item3 | 0.793 |  |  |
|  | item4 | 0.81 |  |  |
|  | item5 | 0.691 |  |  |
|  | item6 | 0.721 |  |  |
|  | item7 | 0.824 |  |  |
|  | item8 | 0.856 |  |  |
|  | item9 | 0.648 |  |  |
|  | item10 | 0.669 |  |  |
|  | item11 | 0.793 |  |  |
| team learn-orientation | item1 | 0.882 | 0.984 | 0.85 |
|  | item2 | 0.884 |  |  |
|  | item3 | 0.968 |  |  |
|  | item4 | 0.938 |  |  |
|  | item5 | 0.973 |  |  |
|  | item6 | 0.952 |  |  |
|  | item7 | 0.943 |  |  |
|  | item8 | 0.895 |  |  |
|  | item9 | 0.969 |  |  |
|  | item10 | 0.854 |  |  |
|  | item11 | 0.875 |  |  |
| team innovation performance | item1 | 0.722 | 0.921 | 0.66 |
|  | item2 | 0.788 |  |  |
|  | item3 | 0.908 |  |  |
|  | item4 | 0.795 |  |  |
|  | item5 | 0.818 |  |  |
|  | item6 | 0.832 |  |  |

2. Add reliability of each construct/variable

Our response: Thanks very much for your helpful suggestions. The Cronbach's alpha for all constructs is between 0.919 and 0.984, which has been reported in the variable Measure chapter. Moreover, according to Table above, the composite reliability (CR) of variables is between 0.921 and 0.984, which is in line with the recommendations of scholars. Based on the above analysis results, we believe that the reliability of the measurement results is acceptable.
3. Add Average variance extracted and composite reliability

Our response: Thanks very much for your helpful suggestions. We have added a description of the CR and AVE results in the data analysis chapter. You can also find specific numerical values in the table above.
4. Discriminant validity is missing (Fornell Larcker criteria or HTMT ratios can be sufficient)

Our response: Thanks very much for your helpful suggestions. We used Fornell-Larcker criteria to test the discriminative validity of variables, as follows:

We employed the Fornell-Larcker criterion to assess construct discriminant validity. The square root of AVEs (Average Variance Extracted) ranged from 0.702 to 0.922, while correlation coefficients between the variables ranged from 0.004 to 0.615. In all cases, the square root of AVEs exceeded the correlation coefficients between variables, providing confirmation of discriminant validity (Line 389-392).
5. Enhance discussion section and compare hypotheses with past studies

Our response: Thank you for your suggestions, and we apologize for the insufficiency in the content of the discussion section. We have restructured the discussion section, including a comparison of the data analysis results with existing research. We have reaffirmed the positive impact of the high-level team learning orientation and TPSRB interaction on team innovation performance, which aligns with previous research (Hirst, 2009). Additionally, we have provided an explanation for the non-establishment of the mediated effect under the premise of low-level team learning orientation. The specific content is as follows:

The results of this study reveal an important phenomenon that team pro-social rule breaking can stimulate individuals to play more active thinking and enhance their creative effort in teams with high level learning orientation. However, a noteworthy implication of our analysis is that in teams with low level learning orientation, certain behaviors may not trigger team members' reflective thinking. Learning orientation, serving as a crucial team condition, dictates the importance individuals place on team behavior and their subsequent responses. These research results align with the findings of Hirst (2009) and other scholars on team learning orientation. A learning-oriented team style makes individuals more attuned to team behavior trends, prompting them to adjust their behaviors accordingly (Hirst, 2009). Additionally, in the Chinese context, organizations often emphasize employee obedience and conformity (Farh, 2000), and in teams with low learning tendency, pro-social rule breaking behaviors are more likely to be regarded as disrespectful behaviors to organizational rules. This can further incite negative feedback from employees regarding such behaviors, without necessarily prompting reflection on the existing work mode.

Hirst G, Van Knippenberg D, Zhou J. A cross-level perspective on employee creativity: Goal orientation, team learning behavior, and individual creativity[J]. Acad manage j, 2009, 52(2): 280-293.

Farh J L, Cheng B S. A cultural analysis of paternalistic leadership in Chinese organizations[M]//Management and organizations in the Chinese context. London: Palgrave Macmillan UK, 2000: 84-127.
6. Add meaningful information in conclusion section

Our response: Thank you for your suggestions. We added the discussion of the research results in the conclusion chapter, and on the basis of summarizing the research results, we emphasized the positive effect of the research on improving team creativity. At the same time, it also puts forward management practice measures based on the research results, the specific contents are as follows:

Our research provides a theoretical basis for management to enhance team creativity. Managers often need to manage teams in complex organizational environments to maintain team innovation efforts. Our research provides feasible solutions to solve this problem, and confirms the promotion effect of the combination of team learning orientation and team pro-social rule breaking on team innovation. The management should be more rational about the positive significance of the team's pro-social rule breaking to the organization, and should enhance the organization's learning initiative and increase employees' self-reflection through active efforts.

Reviewer #3: • The central premise of the paper is built on the relationship between team prosocial rule breaking with innovation performance which hasn’t been checked before however the relationship between team reflexivity and innovation has already been established by extant research that limits the theoretical contributions and novelty of this article. Similarly extant research has studied and found positive relationship between team learning goal orientation and team reflexivity.

Our response: Thank you for your suggestions. Your suggestions have been helpful in improving the structure of our article, clarifying the shortcomings of existing research, and summarizing the theoretical contributions of this paper. Following your advice, we have reorganized the relevant literature on team reflexivity and team innovation performance, as well as summarized the limitations of related research. On the one hand, although some studies have indicated the positive impact of team reflexivity on organizational innovation performance (Fu, 2021), research on the factors influencing team reflexivity and subsequently promoting organizational innovation performance remains limited to organizational factors and the influence of team member traits, lacking a more diverse range of research perspectives. Based on the theory of social information processing, individuals will adjust their behavior within the organizational environment in which they study (Salancik, 1978). The premise of this adjustment involves the analysis of collected information (Chen, 2013). Team members cannot automatically process information generated by ambiguous behavior of pro-social rule breaking, instead, they use a systematic information processing approach that triggers deeper thinking among team members about whether there are issues with the behavior or the rules. This, in turn, leads to a shared perception within the team. Consequently, team members are capable of critically assessing and reflecting on whether the team's behavior is suitable for the current or intended environment, a process known as team reflexivity. Team reflexivity refers to the process by which team members openly reflect on team goals, strategies, and overall business processes to adapt to changes in the environment (Schippers, 2008). Previous studies have indicated that team reflexivity can encourage employees to develop an understanding of the organization's work goals by observing organizational factors such as the human resource system, leadership styles, and the diversity of team goals, ultimately leading to more innovative behaviors (Wang, 2020; Chen, 2016; Nederveen, 2011; Dayan, 2010). Although research on the influence of team reflexivity on creativity performance has unveiled the mechanism of team reflexivity on individual innovation investment, research on the external factors that trigger team reflexivity has primarily focused on the organizational and individual levels and has not explored the impact of team situational factors on team reflexivity (Hoegl, 2006). As mentioned above, team members interact more frequently during working hours (Ren, 2021; Kozlowski, 2012) and have more consistent work goals and clear work divisions (Cooper, 2019). Consistent behavior patterns within a team, as an important source of organizational information, may be more stimulating for individuals to reflect on work goals than other situational factors. Importantly, this reflective process involves the analysis of the current organizational situation and teamwork. In comparison to well-known psychological processes such as psychological contract breach (Kim, 2018) and leadership identification (DeRue, 2010), this cognitive process can better emphasize the impact of social information processing on employees’ cognition and behavior. Therefore, from the perspective of social information processing theory, this study posits that team factors, such as team pro-social rule-breaking, may stimulate team members’ reflexivity, which will affects team innovation performance further. Conditionally, team reflexivity may serve as the mediating factor linking team pro-social rule-breaking to team innovation performance.

The other hand，according to social information processing theory, the cognition of individuals within the team (e.g., team reflexivity) depends not only on past rule breaking but is also influenced by cues from the environment (Narayan, 2021). As an important team behavior orientation, team learning orientation is an environmental condition with strong information clues among various factors at the team level (Sheng, 2016). Team learning orientation represents a high level of learning commitment and open-mindedness, and encourages team members to identify, confront, and continually correct problems with existing rules or behaviors (Chiu, 2021; Atitumpong, 2018; Sinkula, 1997). Thus, when organizations emphasize learning orientation, their members are more likely to openly question the irrational rules that govern organizational behavior within the team (Alerasoul, 2021), which in turn further enhances the team reflexivity process within the team. Research on the influence of team learning orientation on creativity performance has indicated that organizational learning plays a pivotal role in enhancing organizational innovation capability and knowledge utilization (Patky, 2020; López, 2006). From a human resources perspective, learning orientation work pattern embodies a set of methodologies for disseminating and applying knowledge within an organization, allowing for the transfer of acquired knowledge into the workplace, thus creating capabilities that are both effective and efficient (Song, 2008). The research on organizational learning under various organizational scenarios has expanded scholars' insights into the interaction of team learning orientation and organizational characteristics on organizational output (Lumpkin, 2005). However, scholars' research on the interaction effects between team learning orientation and organizational characteristics in complex organizational contexts on organizational innovation output remains limited (Alerasoul, 2021). Existing studies cannot adequately explain whether an organizational atmosphere emphasizing learning can effectively stimulate individuals' deep understanding of organizational characteristics and subsequent organizational behaviors. For responding to the academic community's call for team learning-oriented research (Alerasoul, 2021; Patky, 2020; López, 2006), we introduce team learning orientation as the boundary condition. The result will extend the research boundaries related to pro-social rule-breaking and team learning orientation.

Kozlowski, S. W. J., & Bell, B. S. Work Groups and Teams in Organizations. Handbook of Psychology, Second Edition. Wiley: New York, 2012, pp. 412-469.

Ren, S., Wang, Z., & Collins, N. T. The joint impact of servant leadership and team-based HRM practices on team expediency: the mediating role of team reflexivity. Pers Rev 2021, Vol.50: 1757-1773. doi:10.1108/PR-07-2020-0506.

Salancik, G. R., & Pfeffer, J. A Social Information Processing Approach to Job Attitudes and Task Design. Admin Sci Quart 1978, 23(2), 224-253. doi:10.2307/2392563.

Chen, Z., Takeuchi, R., & Shum, C. A social information processing perspective of coworker influence on a focal employee. Organ Sci 2013, 24(6), 1618-1639. doi: 10.1287/orsc.2013.0820.

Cooper, B., Wang, J., Bartram, T., & Cooke, F. L. Well‐being‐oriented human resource management practices and employee performance in the Chinese banking sector: The role of social climate and resilience. Hum Resour Manage-Us 2019, 58(1), 85-97.

Schippers, M. C., Den Hartog, D. N., Koopman, P. L., & van Knippenberg, D. The role of transformational leadership in enhancing team reflexivity. Hum Relat 2008, 61(11), 1593-1616. doi:10.1177/0018726708096639.

Chen, S., Zhang, G., Zhang, A., & Xu, J. Collectivism-oriented human resource management and innovation performance: An examination of team reflexivity and team psychological safety. J Manage Organ 2016, 22(4), 535. doi:10.1017/jmo.2015.50.

Wang, Z., Ren, S., Chadee, D., Liu, M., & Cai, S. Team reflexivity and employee innovative behavior: the mediating role of knowledge sharing and moderating role of leadership. J Knowl Manag 2020, 25(6): 1619-1639. doi: 10.1108/JKM-09-2020-0683.

Nederveen Pieterse, A., van Knippenberg, D., & van Ginkel, W. P. Diversity in goal orientation, team reflexivity, and team performance. Organ Behav Hum Dec 2011, 114(2), 153-164. doi:10.1016/j.obhdp.2010.11.003.

Dayan, M., & Basarir, A. Antecedents and consequences of team reflexivity in new product development projects. J Bus Ind Mark 2010, 25(1), 18-29. doi:10.1108/08858621011009128.

Hoegl M, Parboteeah K P. Team reflexivity in innovative projects[J]. R&d Management, 2006, 36(2): 113-125.

Kim T T, Karatepe O M, Lee G. Psychological contract breach and service innovation behavior: psychological capital as a mediator[J]. Service Business, 2018, 12: 305-329.

DeRue D S, Ashford S J. Who will lead and who will follow? A social process of leadership identity construction in organizations[J]. Academy of management review, 2010, 35(4): 627-647.

Narayan, S., Sidhu, J. S., & Volberda, H. W. From attention to action: The influence of cognitive and ideological diversity in top management teams on business model innovation. J Manage Stud 2021, 58(8), 2082-2110. doi: 10.1111/joms.12668.

Sheng, M. L., & Chien, I. Rethinking organizational learning orientation on radical and incremental innovation in high-tech firms. J Bus Res 2016, 69(6), 2302-2308. doi:10.1016/j.jbusres.2015.12.046.

Sinkula, J. M., Baker, W. E., & Noordewier, T. A framework for market-based organizational learning: Linking values, knowledge, and behavior. J Acad Market Sci 1997, 25(4), 305. doi:10.1177/0092070397254003.

Atitumpong, A., & Badir, Y. F. Leader-member exchange, learning orientation and innovative work behavior. J Workplace Learn 2018, 30(1): 32-47. doi: 10.1108/JWL-01-2017-0005.

Chiu, C. Y., Lin, H. C., & Ostroff, C.. Fostering team learning orientation magnitude and strength: Roles of transformational leadership, team personality heterogeneity, and behavioural integration. J Occup Organ Psych 2021, 94(1), 187-216. doi: 10.1111/joop.12333.

Alerasoul, S. A., Afeltra, G., Hakala, H., Minelli, E., & Strozzi, F. Organisational learning, learning organisation, and learning orientation: An integrative review and framework. Hum Resour Manage R 2021, 32(3):100854. doi: 10.1016/j.hrmr.2021.100854.

Patky J. The influence of organizational learning on performance and innovation: a literature review[J]. Journal of Workplace Learning, 2020, 32(3): 229-242.

López S P, Peón J M M, Ordás C J V. Human resource management as a determining factor in organizational learning[J]. Management Learning, 2006, 37(2): 215-239.

Song J H, Chermack T J. A theoretical approach to the organizational knowledge formation process: Integrating the concepts of individual learning and learning organization culture[J]. Human Resource Development Review, 2008, 7(4): 424-442.

Lumpkin G T, Lichtenstein B B. The role of organizational learning in the opportunity–recognition process[J]. Entrepreneurship theory and practice, 2005, 29(4): 451-472.

Fu, N., Flood, P. C., Rousseau, D. M., & Morris, T. Resolving the individual helping and objective job performance dilemma: The moderating effect of team reflexivity. J Bus Res 2021, 129, 236-243. doi: 10.1016/j.jbusres.2021.02.058.

• The relationship between Team prosocial rule breaking and Team reflexivity has not been studied before and auhor has proposed the moderating effect of learning orientation without establiblishing the prime hypothesis between Team prosocial rule breaking and Team reflexivity. I suggested here to first establish and justify the relationship between these two variables with soild theoretical argumentation in separate hypothesis and then check the moderation effect.

Our response: Thanks very much for your helpful suggestions. According to the theoretical framework of this paper, the relationship between team pro-social rule breaking and team reflexivity should not be significant, for the following reasons.

First, team pro-social rule breaking is a behavior that breaks the rules of the organization. Although it is in the interests of the organization, such behavior has certain risks, previous studies have also pointed out that pro-social rule breaking negatively affect the work performance evaluated by others (Morrison, 2006), so it may not directly lead to the emergence of team reflexivity without taking into account the organizational context; Second, Following this logic, under the framework of social information processing theory, we believe that the positive relationship between team pro-social rule breaking and team reflexivity can be significant only if a boundary condition effectively eliminate or reduce such risks; Third, team learning orientation is the team members' consistent perception of the organizational learning atmosphere (Chiu, 2021), a high level of team learning orientation indicates more learning commitment, open-mindedness, and shared vision (Alerasoul, 2021; Patky, 2020). Individuals can perceive the organization's emphasis on learning through the team's daily behavior, and the team's behavioral orientation also implies the organization's tolerance for individual work innovation (López, 2006). Open-mindedness reflects the organization's questioning of routine behaviors and conventions, and the organization is more willing to explore new organizational processes by attempting to overturn old work methods (Lord, 2015). Learning commitment and open-mindedness affect team and individual learning motivation through organizational behavior, and shared vision is the degree to which the organization establishes universally recognized learning-oriented values. A high level of shared vision can promote individuals in the team to form a belief in continuous learning and have a greater impact on the team and individual learning directions. Overall, a high level of team learning orientation means that the team can influence individual learning processes and goals through learning methods. Individuals can perceive a more learning-oriented atmosphere in their interactions with the team, which emphasizes the exploration and acceptance of new things. This atmosphere also has the driving force to improve existing work methods. In this organizational context, pro-social rule breaking behavior may stimulate individuals to think more about the rationality of inherent work processes and attempts, and this attempt is tolerated and encouraged by the organization. Therefore, based on the above discussion, we first construct a moderating relationship, emphasizing the positive impact of team pro-social rule breaking on team reflexivity under the premise of team learning orientation; Four, similar model assumptions are more common in variables that exhibit both positive and negative aspects, such as perceived overqualification, which reflects an individual's overall assessment of the difference between their own qualifications and organizational requirements. Previous research has confirmed its dual impact on job performance . For instance, Erdogan (2009), in exploring the negative relationship between perceived overqualification and job satisfaction, did not discuss the direct relationship between them. Instead, the author directly proposed the negative effect of the interaction between perceived overqualification and empowerment on job satisfaction by demonstrating the moderating role of empowerment. Similarly, the author also directly argued the impact of the interaction between perceived overqualification and empowerment on intentions to remain and voluntary turnover. Deng (2016) introduced interpersonal influence as a moderating factor and verified the impact of the interaction between perceived overqualification and interpersonal influence on employee social acceptance. This study did not propose a direct relationship between perceived overqualification and employee social acceptance but assumed that the degree of interpersonal influence could affect the extent and nature of the impact of perceived overqualification on employee social acceptance. Similarly, Huang (2009) revealed the moderating role of team learning orientation in task conflict and relationship conflict. Teams with a high level of learning orientation could extract more diagnostic information from task conflicts, thus reducing individuals' judgments of others' inadequate abilities and, consequently, reducing the likelihood of relationship conflicts. The author did not separately examine the relationship between task conflict and relationship conflict; Five, this way of presenting the hypothesis also highlights the practical value of management. When organizations face team behaviors with dual aspects, it becomes even more crucial to shape their organizational culture and design situational contexts that emphasize the positive aspects of these behaviors while mitigating their adverse effects on the organization. To foster a positive perception of team pro-social rule-breaking among individuals, management needs to allocate more resources to cultivate a team learning environment. With such an environment in place, team members can engage in self-reflection regarding their own work patterns in response to others' pro-social rule-breaking behavior. This, in turn, enables them to enhance their individual and team innovation performance.

Lord M. Group learning capacity: the roles of open-mindedness and shared vision[J]. Frontiers in psychology, 2015, 6: 150.

Erdogan B, Bauer T N. Perceived overqualification and its outcomes: The moderating role of empowerment[J]. Journal of applied psychology, 2009, 94(2): 557.

Deng H, Guan Y, Wu C H, et al. A relational model of perceived overqualification: The moderating role of interpersonal influence on social acceptance[J]. Journal of Management, 2018, 44(8): 3288-3310.

Huang J C. Unbundling task conflict and relationship conflict: The moderating role of team goal orientation and conflict management[J]. International Journal of Conflict Management, 2010, 21(3): 334-355.

• At numerous places author had claimed blunt statemt without proper refernance. E.g “Ac-cording to the social information processing theory, as an individual capable of self-regulation, an employee's subsequent behavior not only depends on the past behavior, but also is influenced by environmental cues.(ref ???) Following this logic, individuals' subsequent responses to collective team violations can be influenced by team environmental cues such as team learning orientation.(ref???) Team learning orientation is a series of values that influence the creation and utilization of knowledge by a team and shape the learning atmosphere of a team.(ref???)” requires strong literature support.
Our response: it is really a giant mistake to the whole quality of our article. We feel sorry for our carelessness. We have thoroughly reviewed the entire manuscript once again. This included two rounds of proofreading by two PhD scholars from our research team and one native English-speaking expert to ensure that the errors you mentioned do not appear. Additionally, we have organized the citations throughout the manuscript and added references to the introduction, hypotheses, and subsequent chapters to enhance the theoretical support for the research content.
• This study has defined multiple ideas in one frame without solid justification and theoretical understanding. The autor need to rethink the ideas and frame them in a structure way with solid argumentaion.

Our response: thank you for your suggestions on this paper. The research framework of this paper is based on the Social Information Processing Theory, emphasizing that TPSRB, as a trigger for team cognition, can further promote an increase in team innovation performance. The Social Information Processing Theory posits that individuals' activities and behaviors do not occur in a vacuum but are typically influenced by complex and ambiguous social contexts (Salancik and Pfeffer, 1978). The social environment in which individuals find themselves provides various pieces of information that influence their attitudes and behaviors. Individuals' interpretation of this information determines their subsequent attitudes and behaviors. Under this theoretical framework, we further emphasize the guiding role of the Social Information Processing Theory on the model and reorganize the research approach. In the introduction chapter, we analyze the impact of TPSRB on team reflexivity and team innovation performance. Through an analysis of existing research, we find that TPSRB reflects the prosocial characteristics of team behavior, but its external violation adds a dual nature to such behavior. These behavioral characteristics highlight the difference between prosocial rule-breaking behavior and conventional organizational behavior. This difference, within a team-supportive learning environment, may serve as a social information cue to trigger employees' reflection on the true purpose of team work and their own work styles. Furthermore, it may encourage individuals to enhance team innovation performance by changing work patterns or other organizational behaviors. In line with this approach, we further elaborate on our hypotheses in the hypothesis formulation chapter. First, we summarize the characteristics of team behavior by reviewing existing research, emphasizing that prosocial rule-breaking behavior at the team level can aggregate within the team. In other words, the prosocial rule-breaking behavior of individuals within the team can 'infect' others and may result in individuals forming a consistent view of team behavioral characteristics. High levels of team prosocial rule-breaking behavior reflect employees' greater concern for the welfare of social roles. When faced with the interests of others and organizational rules, such teams may exhibit more prosocial behavior. Second, we analyze the measurement of team prosocial rule-breaking behavior based on existing research. According to previous research, team behavior can be referred to through various means, such as the mean, extreme values, or other methods of evaluating individual behavior within the team. In the context of this study, team prosocial rule-breaking behavior, as a shared individual cognitive viewpoint, can be measured using the mean. Furthermore, with a clear understanding of the structure and measurement of team prosocial rule-breaking behavior, we analyze the interaction effects of TPSRB and team learning orientation on team reflexivity and team innovation performance based on the Social Information Processing Theory. We put forward specific hypotheses. Through these structural modifications, we aim to enhance the readability of the paper, strengthen its logical flow, and lay the foundation for further clarification of the positive impact of team prosocial rule-breaking behavior on organizations.

Salancik, G. R., & Pfeffer, J. A Social Information Processing Approach to Job Attitudes and Task Design. Admin Sci Quart 1978, 23(2), 224-253. doi:10.2307/2392563.

• This study is backed by numerous theories and multiple conflicting ideas. The author may highlight the role of overarching theory to explain the proposed relationships in all hypothesis.

Our response: Thank you for your suggestions. We fully agree with your assessment, and through a comprehensive review and rewrite of the manuscript, we have emphasized the contribution of the Social Information Processing Theory to the model and hypotheses multiple times.

First, regarding Hypothesis 1, we believe that the interaction between team learning orientation and team pro-social rule breaking provides a prerequisite for triggering team reflexivity. Social Information Processing Theory suggests that the complex and diverse organizational environment increases employees' confusion about the true intentions of the organization (Atitumpong, 2018). To resolve these ambiguities, individuals will analyze unique factors in the organizational context to deepen their understanding of the organization (Chiu, 2021). Therefore, we argue that in the context of high team learning orientation, organizations encourage employees to deepen their understanding of organizational work goals through learning and encourage employees to optimize their work patterns through learning. Once employees perceive the organization's work intentions, they will pay more attention to unique behaviors within the organization, such as team prosocial rule-breaking behavior. The interaction between team pro-social rule breaking behavior and team learning orientation serves as a social information cue influencing individual work attitudes and cognition. Existing research indicates that the interpretation of organizational behavior affects employee work attitudes and work identification (Alerasoul, 2021; Patky, 2020). The interaction between team pro-social rule breaking behavior and team learning orientation will promote employees' perception of the organization's prosocial nature and tolerance (Lumpkin, 2005). In a learning and imitation-promoting environment, individuals will place greater importance on the differences between organizational prosocial rule-breaking behavior and regular organizational behavior. They will analyze trends in organizational behavior and real purposes, leading to increased reflection on their own work behavior and work outcomes (van Ginkel, 2009). This reflection process may involve discovering discrepancies between their own work purposes and organizational goals, as well as differences in their work approaches compared to team members.

Second, regarding Hypothesis 2, we believe that team reflexivity will promote the improvement of team innovation performance. Social Information Processing Theory emphasizes that individuals' understanding and interpretation of the situation can guide their related behavior (Lemerise, 2000). Through team reflexivity, employees will gain a clearer understanding of the organization's work intentions, which helps teams innovate their plans, actions, and other processes to align more closely with the ever-changing circumstances (Chen, 2013). Furthermore, high levels of team reflexivity encourage employees to enhance their innovative thinking and decision-making quality through internal discussions, reduce differences in employee task understanding, help employees develop constructive thinking, identify critical work factors, and share valuable insights and new knowledge through internal discussions (Ren, 2021). In summary, based on Social Information Processing Theory, team reflexivity promotes employees' understanding of the organization's true intentions, strengthens employees' innovative thinking, and enhances their innovation performance. Please refer to the specific revisions in the manuscript on lines 146-157 and 200-208.

Atitumpong, A., & Badir, Y. F. Leader-member exchange, learning orientation and innovative work behavior. J Workplace Learn 2018, 30(1): 32-47. doi: 10.1108/JWL-01-2017-0005.

Chiu, C. Y., Lin, H. C., & Ostroff, C.. Fostering team learning orientation magnitude and strength: Roles of transformational leadership, team personality heterogeneity, and behavioural integration. J Occup Organ Psych 2021, 94(1), 187-216. doi: 10.1111/joop.12333.

Alerasoul, S. A., Afeltra, G., Hakala, H., Minelli, E., & Strozzi, F. Organisational learning, learning organisation, and learning orientation: An integrative review and framework. Hum Resour Manage R 2021, 32(3):100854. doi: 10.1016/j.hrmr.2021.100854.

Patky J. The influence of organizational learning on performance and innovation: a literature review[J]. Journal of Workplace Learning, 2020, 32(3): 229-242.

Lumpkin G T, Lichtenstein B B. The role of organizational learning in the opportunity–recognition process[J]. Entrepreneurship theory and practice, 2005, 29(4): 451-472.

van Ginkel, W., Tindale, R. S., & van Knippenberg, D. Team reflexivity, development of shared task representations, and the use of distributed information in group decision making. Group Dyn-Theor Res 2009, 13(4), 265. doi:10.1037/a0016045.

Lemerise E A, Arsenio W F. An integrated model of emotion processes and cognition in social information processing[J]. Child development, 2000, 71(1): 107-118.

Chen, Z., Takeuchi, R., & Shum, C. A social information processing perspective of coworker influence on a focal employee. Organ Sci 2013, 24(6), 1618-1639. doi: 10.1287/orsc.2013.0820.

Ren, S., Wang, Z., & Collins, N. T. The joint impact of servant leadership and team-based HRM practices on team expediency: the mediating role of team reflexivity. Pers Rev 2021, Vol.50: 1757-1773. doi:10.1108/PR-07-2020-0506.

• Clarity is required regarding the positioning of the article and its theoretical contributions. All hypothesized relationship needs proper references and theoratica argumentation like in H2.

Our response: Thank you for your suggestions. We sincerely appreciate the valuable comments. We rearranged the gaps in the research on TPSRB, team reflexivity and team learning orientation, and re-wrote the theoretical contribution part of the introduction chapter, and revised the content of the theoretical contribution chapter in turn (Line 119-137, 483-498). We have also modified the contents of hypothesis 1 and hypothesis 3.

On hypotheses 1, we restructured the construct to make the logical reasoning of Hypothesis 1 more reasonable. Firstly, we analyzed the positive role of a high level team learning orientation in promoting individuals' processing of organizational information. Under this premise, individuals may have a stronger motivation to analyze the organizational true intent reflected by team pro-social rule breaking and continuously address behavioral issues. Teams with a learning orientation hold more positive views of team pro-social rule-breaking and are willing to establish trust relationships with others through sharing viewpoints. Based on this, we have rewritten Hypothesis 1, as seen in lines 165 to 196. Additionally, we have included the following citation to support Hypothesis 1.

[53]Zhu S, Song J, Hazen B T, et al. How supply chain analytics enables operational supply chain transparency: An organizational information processing theory perspective[J]. International Journal of Physical Distribution & Logistics Management, 2018, 48(1): 47-68.

[54]Jyoti J, Dev M. The impact of transformational leadership on employee creativity: the role of learning orientation[J]. Journal of Asia Business Studies, 2015, 9(1): 78-98.

[55]Baker, W. E., & Sinkula, J. M. The Synergistic Effect of Market Orientation and Learning Orientation on Organizational Performance. J Acad Market Sci 1999, 27(4), 411-427. doi:10.1177/0092070399274002

[56]Pearsall, M. J., & Venkataramani, V., Overcoming asymmetric goals in teams: The interactive roles of team learning orientation and team identification. J Appl Psychol 2015, 100(3), 735-748. doi: 10.1037/a0038315.

[57]Harvey, J. F., Johnson, K. J., Roloff, K. S., & Edmondson, A. C. From orientation to behavior: The interplay between learning orientation, open-mindedness, and psychological safety in team learning. Hum Relat 2019, 72(11), 1726-1751. doi: 10.1177/0018726718817812.

[58]Koopman, J., Scott, B., Matta, F., Conlon, D., & Dennerlein, T. Ethical Leadership as a Substitute for Justice Enactment: An Information-Processing Perspective The dark side of empowering leadership. J Appl Psychol 2019, 104(9): 1103-1116. doi:10.1037/apl0000403.

[59]Vadera, A. K., Pratt, M. G., & Mishra, P. (2013). Constructive Deviance in Organizations:Integrating and Moving Forward. J Manage 2013, 39(5), 1221-1276. doi:10.1177/0149206313475816.

[60]Baharom M N, Sharfuddin M, Iqbal J. A systematic review on the deviant workplace behavior[J]. Review of Public Administration and Management, 2017, 5(3): 1-8.

[61]Cohen, A., & Ehrlich, S. Exchange variables, organizational culture and their relationship with constructive deviance. Manag Res Rev 2019, 42(12), 1423-1446. doi:10.1108/MRR-09-2018-0354.

[62]Fu, N., Flood, P. C., Rousseau, D. M., & Morris, T. Resolving the individual helping and objective job performance dilemma: The moderating effect of team reflexivity. J Bus Res 2021, 129, 236-243. doi: 10.1016/j.jbusres.2021.02.058.

[63]Boone L W, Makhani S. Five necessary attitudes of a servant leader[J]. Review of Business, 2012, 33(1).

[64]Bunderson J S, Sutcliffe K M. Management team learning orientation and business unit performance[J]. Journal of applied psychology, 2003, 88(3): 552.

[65]Breugst N, Preller R, Patzelt H, et al. Information reliability and team reflection as contingencies of the relationship between information elaboration and team decision quality[J]. Journal of Organizational Behavior, 2018, 39(10): 1314-1329.

On hypotheses 3, social information processing theory explains the impact of organizational factors on employee behavior. Given the context of this study, we believe that the interaction between team pro-social rule breaking and team learning orientation, as significant organizational cues, stimulates employees to re-cognize their expectations and job requirements within the organization. This process internalizes through team reflexivity, becoming a driving force for employees' innovation in their own work. Existing research indicates that social information processing theory explains the pathway through which organizational behavior influences employee behavior. Individual expectations of their own work patterns are formed through the analysis of continuous or isolated organizational behaviors, guiding them to improve their work output accordingly. This behavioral pathway is influenced by more organizational behavior in complex organizational environments since employees face greater difficulties in understanding organizational intentions. Consequently, they rely more on information provided by the organization to analyze its needs. Team learning orientation enhances the learning motivation of individuals within the organization. Employees are more willing to improve their work performance by learning from the behavior of others. A high level of team learning orientation promotes individual understanding of team pro-social rule-breaking through internal reflection and discussion. Through concentrated internal discussions, individuals within the team can form a consensus on innovative work patterns, further guiding the improvement of team innovation performance. Based on the above analysis, we believe that team learning orientation moderates the indirect relationship between team pro-social rule breaking and team innovation performance through team reflection. The higher the team learning orientation, the stronger the indirect relationship. Based on this, we have rewritten Hypothesis 3, as seen in lines 229 to 248.

• The paper need restructuring of ideas and must be in acceptable language as I have seen numerous grammatical and typo mistakes. E.g

• Line 42: “…workplace when they in-novate”- the mistake of putting a hyphen in a word has been made several times throughout the paper.
• Line 43: “…subjects of innovation activity”- drivers is innovation activity is a more appropriate phrase.
• Line 43: “…employees inevitably challenge and break with practices”- It should be break away.
• Line 63: “…and this process that is known as team reflexivity”-this is a grammatically incorrect or an incomplete sentence.
• Line 68: “In other words, team members accumulate and evaluate the information conveyed by the contradictions in past actions by observing themselves or the environment in order to make adaptations and changes to the environment”. This sentence is very ambiguous and does not make the purpose of writing the sentence clear to the reader.
• Line 86: “The theoretical model framework of this study was depicted in Figure 1 as follows”- this is a grammatically incorrect sentence.
• Line 88: “First, we attempt to identify the types of pro-social rule breaking at the team level fill a notable perspective gap.” This is a grammatically incorrect sentence.
• Line 89: “Previous research has focused merely on pro social rule breaking at the individual lev-el and has not yet focused on the team level.” This sentence requires citations.
• Line 102: Previous research has focused merely on pro social rule breaking at the individual lev-el and has not yet focused on the team level. This is grammatically incorrect sentence.
• Line 104, 105, 106, 107: Firstly, team members are a crucial factor of work environment, team members are able to inform certain activities and help others shape beliefs about what should do or not do [9, 25]. People can signal from the fact that colleagues act pro-social rule breaking behavior for the benefit of the organization or stakeholders.
• The above sentences are grammatically incorrect and ambiguous, failing to make any meanings.
• Line 109-110: Secondly, pro-social rule breaking refers to help organizations, colleagues, or customers with altruistic intentions, even if leading to negative results. This sentence is grammatically incorrect.
• Line 111: “…and then likely to imitate in the future.” This is grammatically incorrect.
Our response: thank you for your suggestions regarding the structure and grammar of this paper. We also apologize for any frustration caused by our poor writing, which may have hindered your understanding of the paper's content. As per your request, we have reexamined the paper, extensively reviewed and revised it for grammar issues, and rewritten sentences that raised concerns. To ensure the accuracy of the paper, we engaged an expert in the field to proofread the entire document. This expert is a native English speaker with extensive experience in writing scientific papers and provided constructive feedback on the paper's structure, writing conventions, grammar, and word usage. We hope that these modifications will enhance the quality and readability of the paper.
• I have serious concerned with the methodological section of this paper as the validity of construct has not reported in paper. Author has just reported main variable context results and not compare every variable with other in factors model. Bootstrap sample size is missing, similarly I have not found ICCI,ICC2 or RWG test. The author also has not reported moderation graph. Apart from that I also suggest to report the values of simple slope analysis in interpretation with justification that what happened at high and low levels.

Our response: thank you for your suggestions. The construct validity of the variables are also examined before testing the hypotheses. A series of confirmatory factor analyses (CFA) was conducted to examine the distinctiveness of our study variables based on chi-square statistics and fit indices of RMSEA, CFI and TLI. As shown in Table below, the fit indices support that the hypothesized 4-factor model of team pro-social rule breaking, team learning orientation, team reflexivity, and team innovation performance, χ 2 = 173.277, df = 48; RMSEA = 0.083; CFI = 0.972; SRMR=0.036 and TLI = 0.962, yielded a better fit to the data than the three-factor, two-factor and one-factor models. These CFA results also provide support for the distinctiveness of the four study variables for subsequent analyses. See lines 372 to 395 for details.

| **Model** | ***χ^2^*** | ***df*** | ***χ^2^/ df*** | **TLI** | **CFI** | **RMSEA** | **SRMR** | $\Delta$***χ^2^*** |
| --- | --- | --- | --- | --- | --- | --- | --- | --- |
| four-factor model | 173.277 | 48 | 3.610 | 0.962 | 0.972 | 0.083 | 0.036 |  |
| three-factor model 1 | 998.734 | 51 | 19.583 | 0.730 | 0.791 | 0.221 | 0.179 | 825.457 |
| three-factor model 2 | 1541.952 | 51 | 30.234 | 0.575 | 0.671 | 0.277 | 0.249 | 1368.675 |
| three-factor model 3 | 1776.177 | 51 | 34.827 | 0.508 | 0.620 | 0.298 | 0.260 | 1602.900 |
| three-factor model 4 | 1448.751 | 51 | 28.407 | 0.601 | 0.692 | 0.268 | 0.201 | 1275.474 |
| three-factor model 5 | 1651.653 | 51 | 32.385 | 0.543 | 0.647 | 0.287 | 0.202 | 1478.376 |
| three-factor model 6 | 1173.375 | 51 | 23.007 | 0.680 | 0.753 | 0.240 | 0.095 | 1000.098 |
| two-factor model 1 | 2359.961 | 53 | 44.528 | 0.367 | 0.491 | 0.338 | 0.295 | 2186.684 |
| two-factor model 2 | 2592.625 | 53 | 48.917 | 0.303 | 0.440 | 0.354 | 0.303 | 2419.348 |
| two-factor model 3 | 2967.082 | 53 | 55.983 | 0.200 | 0.357 | 0.379 | 0.309 | 2793.812 |
| two-factor model 4 | 1891.810 | 53 | 35.695 | 0.495 | 0.595 | 0.301 | 0.152 | 1718.533 |
| one-factor model | 3749.461 | 54 | 69.434 | 0.004 | 0.185 | 0.423 | 0.337 | 3576.184 |

In the revised manuscript, we have also specified that the sample size for bootstrapping is 20,000. Please refer to line 339 for details. Additionally, we have reported the moderation graph; please see line 413. Furthermore, we have included a simple slope analysis. The simple slope analysis showed that when team members perceived a high level of team learning orientation (+SD), the effect of team pro-social rule breaking on team reflexivity was significant (β= 0.436, 95%CI [0.282, 0.726], excluding 0). When team members perceived low levels of team learning orientation (-SD), team pro-social rule breaking had no significant effect on team reflexivity (β=-0.113, 95% CI [-0.416, 0.117], including 0). The results indicate that the low level of team learning orientation cannot effectively stimulate team reflexivity. One possible explanation is that the low level of team learning orientation leads team members to ignore the behavioral characteristics of team pro-social rule breaking, which is consistent with the previous research results on the impact of team learning orientation on organizational innovation performance. Please see lines 446 to 450 in the revised manuscript for more details.

Finally, we sincerely apologize for the errors made in our manuscript. While collecting questionnaire responses, team pro-social rule breaking was assessed by aggregating employees' pro-social behavior at the individual level to form team-level results. Team reflexivity and team innovation performance are mainly evaluated by the supervisors. However, for the variables of team reflexivity, we mistakenly wrote that they were measured by employee assessments in the manuscript. We deeply regret this mistake and have reanalyzed the research data. We have added a data aggregation section describing the aggregation of pro-social rule breaking from the individual level to the team level. The results showed that the within-group agreement (r_wg_) of team pro-social rule breaking met the study requirements (Average = 0.905; Median = 0.952; Lance et al., 2006; LeBreton & Senter, 2008). Similarly, the intra-class correlation coefficient (ICC (1)) is 0.370, indicating the proportion of variance interpreted by team members (LeBreton and Senter, 2008). To assess the group effect, we also tested the ICC (2) of team pro-social rule breaking, which was 0.669, indicating that the proportion of variance explained at the team level met the study requirements. Taken together, these results support the aggregation of individual scores for all target variables at the team level. Please see lines 384 to 394 in the revised manuscript for more details.

You can access the specific data for this study in <https://dataverse.harvard.edu/dataset.xhtml?persistentId=doi:10.7910/DVN/UASKNB>.

We apologize again for the mistake in the data analysis section.

[NOTE: If reviewer comments were submitted as an attachment file, they will be attached to this email and accessible via the submission site. Please log into your account, locate the manuscript record, and check for the action link "View Attachments". If this link does not appear, there are no attachment files.]

While revising your submission, please upload your figure files to the Preflight Analysis and Conversion Engine (PACE) digital diagnostic tool, <https://pacev2.apexcovantage.com/>. PACE helps ensure that figures meet PLOS requirements. To use PACE, you must first register as a user. Registration is free. Then, login and navigate to the UPLOAD tab, where you will find detailed instructions on how to use the tool. If you encounter any issues or have any questions when using PACE, please email PLOS at [figures@plos.org](mailto:figures@plos.org). Please note that Supporting Information files do not need this step.

 在修改您的提交时，请将您的图形文件上传到飞行前分析和转换引擎(PACE)数字诊断工具https://pacev2.apexcovantage.com/。PACE有助于确保数字符合公共科学图书馆的要求。要使用PACE，您必须首先注册为用户。注册是免费的。然后，登录并导航到UPLOAD选项卡，在这里您将找到有关如何使用该工具的详细说明。如果您在使用PACE时遇到任何问题或有任何疑问，请发送电子邮件至figures@plos.org。请注意，支持信息文件不需要此步骤。
