# Supplementary material for: Team pro-social rule breaking and team innovation performance: An information processing theory perspective
Source: PLoS One. 2024 Jul 11;19(7):e0303800. doi: 10.1371/journal.pone.0303800 (PMC11239028; doi:10.1371/journal.pone.0303800)
Supplement: Attachment — Submitted filename: Response to Reviewers 3-1.0.docx [file pone.0303800.s003.docx]

Thank you to Reviewer 3 for the valuable guidance on the scientific aspects of the manuscript. In response to your queries, the research team engaged in focused discussions, made modifications to relevant content, and highlighted these changes in the manuscript. We hope that our efforts on the manuscript will assist the reviewer in better understanding the revised content. Below is our response to your inquiries, along with prominent explanations of the manuscript changes.

**Reviewer #3: Thank you for addressing the previous highlighted issues but still I have following concerns.The Author has ignored my previous observation regarding the direct relationship between team prosocial rule breaking with innovation performance which hasn’t been checked before. please address this issue by proposing relationship and justify if the results are not significant.**

**Our response:** Thank you for your comments and suggestions on this paper. Based on your suggestions, the research team has proposed the main effect of team pro-social rule breaking on team innovative performance after summarizing the literature on pro-social rule-breaking. The specific content can be found in the following text. Additionally, the modifications related to hypothesis 1 are reflected in the hypothesis formulation, data analysis, and discussion sections of the main text (details are highlighted in yellow). Once again, thank you for your suggestions, which have contributed to enhancing the scientific rigor and logic of this paper.

Hypothetical 1 text:

Researches indicate that pro-social rule breaking, which reflects individuals' autonomy in work and their investment in others' resources and aims to maximize stakeholders' needs, is often related to positive organizational performance [3, 4, 8]. Based on existing research, team pro-social rule breaking may also be a direct factor influencing team innovative performance.

Firstly, pro-social rule breaking indicates that employees, to a certain extent, overlook organizational norms in pursuit of work efficiency [2]. This behavioral characteristic reflects employees' ability to freely choose more appropriate methods to complete tasks when faced with conflicts between behaviors and norms. Research by Chang (2023) and other scholars has indicated that a high level of work autonomy is often associated with team innovation [41]. At the team level, the overt characteristics of pro-social rule-breaking behavior can facilitate more efficient organization of work interactions among colleagues, which will be beneficial for the improvement of team innovative performance [18]. Secondly, pro-social rule breaking also embodies assistance towards colleagues, manifested as a common practice of helping others within the team [2]. Existing research has shown that helping behavior within teams promotes the enhancement of team innovative performance [19]. Additionally, similar to efficiency-oriented pro-social rule-breaking behavior, pro-social rule-breaking behavior towards customers can convey the true purpose of work to individuals and strengthen individuals' awareness of work autonomy [2]. From an observer's perspective, individuals' perceptions of colleagues' pro-social rule-breaking behavior towards customers can provide specific service inspirations, and these cues can provide external perceptual conditions for individual innovation from aspects such as interaction methods and work processes [9], contributing to the improvement of collective innovative performance at the team level. In conclusion, a hypothesis regarding team pro-social rule-breaking behavior and team innovative performance is proposed: H1: Team pro-social rule-breaking behavior positively influences team innovative performance.

H1: Team pro-social rule-breaking behavior positively influences team innovative performance .

• **Generally, we have proposed relationship based on supporting theory and previous literature, but your feedback in response letter has demonstrated that the relationship between team pro-social rule breaking and team reflexivity should not be significant. It means theoretically it is not justifiable so what is the logic behind this framework.**

**Our response:** Thanks for your feedback and suggestions on this paper. Based on the theoretical analysis framework of this paper, there is a causal path relationship between team pro-social rule-breaking behavior and team reflexivity, but it requires situational conditions to be triggered. Reviewing similar existing studies, we believe that team pro-social rule breaking needs to be stimulated by the variable of team learning orientation to positively influence team reflexivity and thus enhance team innovative performance. In other words, team pro-social rule breaking needs specific organizational factors to trigger a tendency towards cognitive change among team members.

In the field of organizational science, research concepts with similar double-edged sword effects generally exhibit such characteristics, such as pro-social rule breaking behavior (which combines altruism and rule-breaking characteristics) and perceived overqualification (POQ, where employees perceive their qualifications to exceed the experiential, educational, and skill requirements of their position, potentially leading to higher job performance but also lower job performance due to misalignment, perceived unfairness, and perceived deprivation mechanisms that may even lead to organizational indignation). Studies on these similar variables indicate that the reasons for the insignificant path relationship between the independent variable of pro-social rule-breaking behavior and the mediating and outcome variables can be divided into two types. The first type is when the independent variable is indeed unrelated to the mediating variable, meaning there is no correlation between the two; the second type is when the independent variable's impact on the mediating variable is multifaceted, meaning there may be both positive and negative effects coexisting. At the data level, these effects may cancel each other out, resulting in no correlation, and only under certain interactive situational factors can the positive effects "emerge."

Fig 1. The interaction effect of the social violation atmosphere and task dependency on the team is negatively influenced by the harmony of the team

Shum (2022) conducted a study on the impact of employees' pro-social safety rule breaking on customer satisfaction. Based on practical theory, the study indicates that employees' pro-social safety violations only have a negative impact on bystanders' service performance evaluations and perceptions of safety when considering the customer's role. In the context of the COVID-19 pandemic, customers' awareness of their role has strengthened their expectations for self-service safety and service quality. Pro-social safety violations, from the bystander's perspective, manifest as service personnel breaking hotel rules, potentially endangering the safety interests of other guests represented by bystanders. Therefore, this behavior can lead to negative evaluations of hotel service performance by bystanders and raise doubts about service safety. The specific research model is illustrated in the diagram below:

Figure 2. The interaction between employee pro-social safety violation and customer role has a negative impact on service performance and perceived safety

The same method of hypothesis presentation is also found in other research areas within organizational behavior studies. Taking POQ as an example, Lobene (2013) highlighted that the interaction effect between mission orientation and POQ negatively impacts turnover intention. When employees have a higher sense of work mission, the negative impact of POQ on turnover intention diminishes. Cheng (2018), based on equity theory, validated the interactive effect between achievement needs and POQ on harmonious passion. Employees with a high need for achievement may be more susceptible to the mismatch between individuals and their work (i.e., being overqualified for their position), leading to a loss of harmonious passion for work. The specific research model is depicted in the diagram below:

Figure 3. The interaction between POQ and achievement need affects harmonious enthusiasm

Deng (2018) directly proposed in a study on the impact of POQ on organizational performance that the interaction effect between interpersonal influence and POQ affects social acceptance. The research findings indicate that the level of interpersonal influence determines the relationship between POQ and social acceptance. When interpersonal influence is high, this relationship is positive; when interpersonal influence is low, this relationship is negative. The data analysis results of two time-lagged studies support this hypothesis, and the examination of correlation confirms that the correlation between POQ and social acceptance is not significant.

Figure 4. The interaction between POQ and interpersonal influence affects social acceptance

Erdogan (2020) pointed out in his study on the impact of POQ on Advice Network Centrality that the moderating effect of P-O fit on the relationship between POQ and Advice Network Centrality only has a significant impact when the P-O fit moderating factor is present. Specifically, when P-O fit is high, the moderating effect has a positive impact on the mediating factor, whereas when P-O fit is low, the moderating effect has a negative impact on the mediating factor. Data analysis results indicate that the correlation between POQ and giving advice is not significant, but the moderating effect of P-O fit on the relationship between POQ and giving advice is significant. The specific research model is illustrated in the following figure:

Figure 5. Interaction between POQ and P-O fit affects OCB and Voice

As mentioned earlier, similar to POQ, the impact of pro-social rule-breaking behavior on organizations and individuals is diverse. In the data analysis phase, it may be observed that the correlation between the independent variable (PSRB) and the mediating variable is not significant. The influence on the mediating variable is achieved only under specific contextual factors. Therefore, drawing from relevant literature, the research team directly proposes the interactive effect of TPSRB and team learning orientation on positive team reflexivity. Additionally, considering the completeness of the paper, based on your suggestion, the research team has added a discussion on the correlation between TPSRB and team reflexivity in the discussion section to enhance the logical coherence of the paper.

Once again, thank you for your suggestions, which have improved the logical flow of this paper.

References:

CAI Y, CHENG J, LI J. Rules can maintain harmony? The influence of team pro-social rule breaking climate on team performance from the perspective of harmony management[J]. Acta Psychologica Sinica, 2022, 54(1): 66.

Shum C, Ghosh A. Safety or service? Effects of employee prosocial safety-rule-breaking on consumer satisfaction[J]. International Journal of Hospitality Management, 2022, 103: 103225.

Lobene E V, Meade A W. The effects of career calling and perceived overqualification on work outcomes for primary and secondary school teachers[J]. Journal of Career Development, 2013, 40(6): 508-530.

Deng H, Guan Y, Wu C H, et al. A relational model of perceived overqualification: The moderating role of interpersonal influence on social acceptance[J]. Journal of Management, 2018, 44(8): 3288-3310.

Erdogan B, Karaeminogullari A, Bauer T N, et al. Perceived overqualification at work: Implications for extra-role behaviors and advice network centrality[J]. Journal of Management, 2020, 46(4): 583-606.

[NOTE: If reviewer comments were submitted as an attachment file, they will be attached to this email and accessible via the submission site. Please log into your account, locate the manuscript record, and check for the action link "View Attachments". If this link does not appear, there are no attachment files.]

While revising your submission, please upload your figure files to the Preflight Analysis and Conversion Engine (PACE) digital diagnostic tool, <https://pacev2.apexcovantage.com/>. PACE helps ensure that figures meet PLOS requirements. To use PACE, you must first register as a user. Registration is free. Then, login and navigate to the UPLOAD tab, where you will find detailed instructions on how to use the tool. If you encounter any issues or have any questions when using PACE, please email PLOS at [figures@plos.org](mailto:figures@plos.org). Please note that Supporting Information files do not need this step.
